# Gene Expression Profiling in Leiomyosarcomas and Undifferentiated Pleomorphic Sarcomas: SRC as a New Diagnostic Marker

**DOI:** 10.1371/journal.pone.0102281

**Published:** 2014-07-16

**Authors:** Rolando A. R. Villacis, Sara M. Silveira, Mateus C. Barros-Filho, Fabio A. Marchi, Maria A. C. Domingues, Cristovam Scapulatempo-Neto, Samuel Aguiar, Ademar Lopes, Isabela W. Cunha, Silvia R. Rogatto

**Affiliations:** 1 Neogene Laboratory, Research Center (CIPE), A. C. Camargo Cancer Center, São Paulo, Brazil; 2 Inter-Institutional Grad Program on Bioinformatics, Mathematics and Statistics Institute, USP - University of São Paulo, São Paulo, Brazil; 3 Department of Pathology, School of Medicine, UNESP - São Paulo State University, Botucatu, São Paulo, Brazil; 4 Molecular Oncology Research Center, Barretos Cancer Hospital, Barretos, São Paulo, Brazil; 5 Department of Pelvic Surgery, A. C. Camargo Cancer Center, São Paulo, Brazil; 6 Department of Pathology, A. C. Camargo Cancer Center, São Paulo, Brazil; 7 Department of Urology, School of Medicine, UNESP - São Paulo State University, Botucatu, São Paulo, Brazil; University of L'Aquila, Italy

## Abstract

**Background:**

Undifferentiated Pleomorphic Sarcoma (UPS) and high-grade Leiomyosarcoma (LMS) are soft tissue tumors with an aggressive clinical behavior, frequently developing local recurrence and distant metastases. Despite several gene expression studies involving soft tissue sarcomas, the potential to identify molecular markers has been limited, mostly due to small sample size, in-group heterogeneity and absence of detailed clinical data.

**Materials and Methods:**

Gene expression profiling was performed for 22 LMS and 22 UPS obtained from untreated patients. To assess the relevance of the gene signature, a meta-analysis was performed using five published studies. Four genes (*BAD*, *MYOCD*, *SRF* and *SRC*) selected from the gene signature, meta-analysis and functional *in silico* analysis were further validated by quantitative PCR. In addition, protein-protein interaction analysis was applied to validate the data. SRC protein immunolabeling was assessed in 38 UPS and 52 LMS.

**Results:**

We identified 587 differentially expressed genes between LMS and UPS, of which 193 corroborated with other studies. Cluster analysis of the data failed to discriminate LMS from UPS, although it did reveal a distinct molecular profile for retroperitoneal LMS, which was characterized by the over-expression of smooth muscle-specific genes. Significantly higher levels of expression for *BAD*, *SRC*, *SRF*, and *MYOCD* were confirmed in LMS when compared with UPS. *SRC* was the most value discriminator to distinguish both sarcomas and presented the highest number of interaction in the *in silico* protein-protein analysis. SRC protein labeling showed high specificity and a positive predictive value therefore making it a candidate for use as a diagnostic marker in LMS.

**Conclusions:**

Retroperitoneal LMS presented a unique gene signature. SRC is a putative diagnostic marker to differentiate LMS from UPS.

## Introduction

Soft Tissue Sarcomas (STS) comprise a heterogeneous group of mesenchymal tumors that represent around 1% of all neoplasms [1], [2].

Diagnosis of these tumors poses a challenge to the pathologist due to their rarity, pleomorphic nature and histologic overlap with numerous sarcoma subtypes [2]. The majority of undifferentiated pleomorphic sarcomas (UPS) demonstrate similar morphology to undifferentiated and pleomorphic tumor subtypes, particularly leiomyosarcoma (LMS) and liposarcoma [1]–[3]. UPS, previously known as malignant fibrous histiocytoma (MFH), represents 17% of STS and has an extremely aggressive pattern of behavior [4], [5]. Leiomyosarcoma accounts for 24% of all STS [4]. In general, Immunohistochemical (IHC) assays for well-differentiated LMS show positivity for actin, desmin, h-caldesmon, transgelin and sirtuin [6]–[8], however, none of these markers specifically differentiate smooth muscle. High-grade LMS shows histological similarities to UPS, which can cause difficulties in the distinction between these neoplasms [6].

The morphological and histopathological similarities between high-grade LMS and UPS are also observed at molecular level. Although copy number alterations have been described as similar in both tumors [9], [10], gains of 1q21.3 have been reported as an independent prognostic marker for shorter survival in patients with LMS [11]. Hierarchical clustering analysis of transcriptomic data for a large series of STS failed to discriminate LMS and UPS. However, tumor subgroups with similar gene expression profiles have been reported [12]–[14]. Studies that combine gene-expression and DNA copy number alterations have also failed to differentiate between these neoplasms [15], however, three novel molecular subtypes of LMS have been described [6], [16], in addition to a genetic signature capable of predicting metastasis in UPS and LMS [17]. More recently, retroperitoneal LMS (LMS-R) has been described as different from LMS of extremities at the molecular level, demonstrating a distinct clinical outcome [18].

In this study, gene expression profiles were evaluated in 44 STS samples obtained from untreated patients prior to surgery, aiming to identify molecular biomarkers.

## Materials and Methods

### Patients

**Figure 1:**
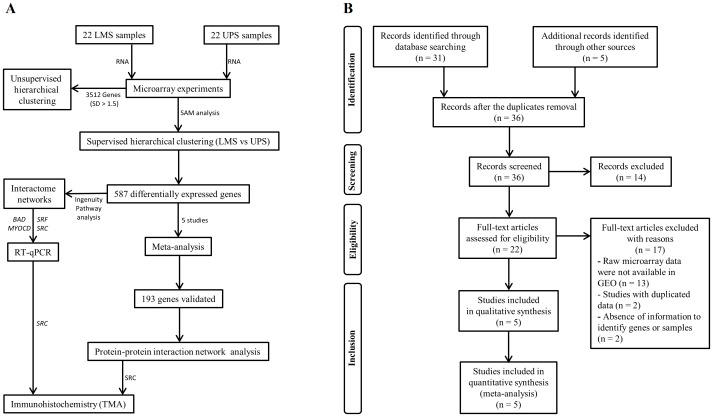
Flow diagrams showing the study design (A) and meta-analysis (B).

Forty-four fresh frozen tissue samples (22 UPS and 22 LMS) were obtained prior to chemotherapy or radiotherapy from 43 patients, at the A.C. Camargo Cancer Center (São Paulo, Brazil) and Barretos Cancer Hospital (Barretos, São Paulo, Brazil), between 2002 and 2010. Two primary tumors (UPS8 and UPS18) were obtained from the same patient. Ninety formalin-fixed paraffin-embedded (FFPE) samples (52 LMS and 38 UPS, which included 12 LMS and 7 UPS evaluated by oligoarrays) were analyzed using IHC assays. Study design is shown in Figure 1A.

This study was approved by the Human Research Ethics Committees of both institutions (A. C. Camargo Cancer Center Protocol 1240/09 and Barretos Cancer Hospital Protocol 302/2010). All patients were advised of the procedures and provided written informed consent. The pathological information of tumor samples (*N* = 44) is summarized in Table 1. Diagnostic criteria were based on the recommendations of the World Health Organization (WHO) [19]. Tumor grade was defined according to the recommendations of the *Federation Nationale des Centres de Lutte Contre le Cancer* (FNCLCC) [20]. An antibody panel for IHC was used to confirm or refute the diagnosis of LMS and UPS (Text S1).

**Table 1:** Pathological features of 22 LMS and 22 UPS samples evaluated by large-scale expression analysis.

Variable	LMS (*N* = 22)	UPS (*N* = 22)
**Sample source**		
Primary tumour	17 (77%)	17 (77%)
Recurrence	5 (23%)	5 (23%)
**Topology**		
Extremity	9 (41%)	11 (50%)
Retroperitoneum	10 (45%)	6 (27%)
Trunk	2 (9%)	2 (9%)
Head and neck	0	2 (9%)
Pelvis	1 (5%)	1 (5%)
**Tumor size**		
<5 cm	5 (23%)	3 (14%)
>5 cm	17 (77%)	19 (86%)
**Histological grade**		
I	4 (18%)	0
II	5 (23%)	0
III	13 (59%)	22 (100%)
**Local recurrence** *		
Yes	8 (36%)	10 (45%)
No	14 (64%)	12 (55%)

*not considered surgery extension of surgical margins.

The median age of patients at diagnosis was 58.8 years (ranging from 4–90 years). Twenty-four out of 43 cases were male. The majority of the patients was treated by surgery (18) or surgery combined with chemotherapy and radiotherapy (10). Distant metastases were identified in 12 patients. The mean follow-up period was 42.1 years (ranging from 1–209 months). In the last follow-up, 28 patients were alive and 15 were dead of disease.

### Transcriptional profiling (oligoarrays)

Total RNA was extracted using Trizol reagent (Invitrogen, Carlsbad, CA, USA) according to the manufacturer's instructions. The RNA quality was assessed using the RNA 6000 Nano Kit on the Agilent 2100 Bioanalyzer platform (Agilent Technologies, Palo Alto, CA, USA). Only samples with RIN (RNA integrity number) >7 were considered. The Two-Color Human GE 4X44K Microarrays platform (Agilent Technologies) was used [21]. A combination of equal amounts of total RNA obtained from 15 different cell lines was used as reference [22]. Hierarchical clustering analysis (HCL) of the most variant probes (standard deviation, SD >1.5) was used to visualize cluster samples. Significance Analysis of Microarray (SAM) was applied to compare LMS and UPS [23]. All experiments were analyzed using the MeV 4.8 software (available at: http://www.tm4.org/mev/). Ingenuity Pathway Analysis (IPA, Ingenuity System Inc, Redwood City, CA, USA) was used to construct interactome networks with the differentially expressed genes between LMS and UPS. The microarray data have been deposited in NCBI's Gene Expression Omnibus (GEO, http://www.ncbi.nlm.nih.gov/geo/) and are accessible through GEO Series accession number GSE49941.

### Meta-analysis of microarray studies

The meta-analysis was conducted according to the Preferred Reporting Items for Systematic Reviews and Meta-Analyses (PRISMA) [24]. The literature search was performed in the PubMed database (http://www.ncbi.nlm.nih.gov/pubmed/) using the following terms: “soft tissue sarcoma”, “leiomyosarcoma”, “undifferentiated pleomorphic sarcoma”, “malignant fibrous histiocytoma” and “gene expression array”. Additional search was identified by manually cross-referencing abstracts and articles published up to November 2013 independently of language, publication year or other limits (Figure 1B). Titles and abstracts were evaluated to identify relevant information and the full texts were archived for analysis. The criteria for inclusion were: (1) The diagnostic of UPS and LMS had to be confirmed and described in the same study; (2) studies with reference standard for the diagnosis of UPS and LMS; (3) studies with raw microarray data available in GEO. Exclusion criteria were: (1) studies with duplicate data reported in other studies; (2) absence of clinical and histological information; (3) studies that were letters, editorials, case reports or case series. Five microarray datasets, containing 309 tumors (134 LMS and 175 UPS), were selected for the final analysis [12], [13], [17], [25], [26]. The ratio of LMS - UPS was calculated individually for each dataset and expression direction (over-expression or down-expression) of each gene was checked and recorded. Differentially expressed genes were submitted to *in silico* protein-protein interaction analysis [27], [28].

### Transcript expression levels using real time quantitative reverse transcription PCR (RT-qPCR)

Expression levels of *BAD*, *SRC*, *SRF* and *MYOCD* were assessed for 13 UPS and 12 LMS (previously analyzed by microarray), which were selected according to the availability of RNA. Primer sequences are provided in Table S1. The reactions were carried out in duplicate as previously described [21]. Only replicates with low variability (ΔCycle quantification <0.5) were considered for analyses. *HMBS, HPRT* and *GAPDH* were selected as reference transcripts [29]. The relative gene expression was calculated according to the Pfaffl method [30].

### Protein expression analysis

A tissue microarray (TMA) was constructed with the Tissue Microarrayer (Beecher Instruments, Silver Springs, USA). Each sample was represented in quadruplicates. IHC was carried out using rabbit polyclonal anti-SRC (Abcam: ab47405, Cambridge, UK) (dilution 1∶200). Positive breast tumor tissue and two negative controls were assessed by IHC. The negative controls were created via omission of the primary antibody and incubation of the slides in PBS, followed by replacement of the primary antibody with normal rabbit serum. The final scores (median of the four scores) were obtained according to staining intensity of the cytoplasm or membrane and were described as negative/weak (score 0–1) or positive (score 2–3). The samples were scored blind with respect to clinical data of the patient.

### Data analyses

Fisher's exact and chi-square tests were used to determine the association between the categorical variables. Overall survival (OS) probability was calculated using the Kaplan-Meier method and the Log Rank test for significance. The Mann Whitney test was used to compare RT-qPCR results. Receiver Operating Characteristic (ROC) curves and classification models designed with Fisher discriminant analysis evaluated the classification properties of markers assessed by RT-qPCR. Statistical analyses were performed using the SPSS 17.0 software (SPSS, Chicago, IL, USA) and GraphPad Prism 5.0 (GraphPad Software Inc., La Jolla, CA).

## Results

### LMS-R as a single entity in gene expression profiling analysis

**Figure 2:**
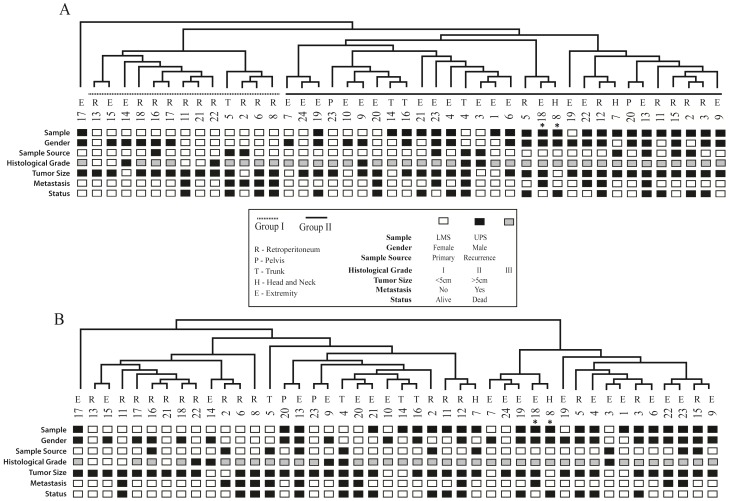
Hierarchical clustering of microarray data. (A) Unsupervised hierarchical clustering (UHCL) for 22 LMS and 22 UPS revealed two global clusters (I and II) and 3,512 genes differentially expressed (standard deviation >1.5). Cluster I included all retroperitoneal LMS (LMS-R), while cluster II contained the majority of the sarcomas of extremities. (B) Supervised hierarchical clustering (SHCL) using the 587 differentially expressed genes from the two-class unpaired Significance Analysis of Microarray (SAM) with FDR <1% between UPS and LMS. LMS-R samples demonstrated the most distinct molecular profile. Samples from the same patient are indicated with an asterisk. Images adapted from the output of the MeV 4.8 software.

Unsupervised hierarchical clustering (UHCL) analysis using 3512 genes (SD >1.5) revealed two clusters (I and II) including 43 samples, with one case (UPS17) clustering separately (Figure 2A). Cluster I was composed of LMS samples only (13) (*P*<0.001). Interestingly, all LMS-R samples (*N* = 10) clustered together (*P*<0.001). One LMS of trunk (LMS5) and two LMS of extremities (LMS14 and LMS15) were also grouped in Cluster I. Cluster II contained the remaining nine LMS from different topologies and 21/22 UPS samples (*P*<0.001). Tumors with histological grade I were detected exclusively in cluster I (*P* = 0.004). No significant difference was found in the comparison between the clusters and patient age (*P* = 0.504), gender (*P* = 0.186), tumor size (*P* = 1.0), presence of metastasis (*P* = 0.460), local recurrence (*P* = 0.332) and overall survival (*P* = 0.392) (data not shown).

Supervised hierarchical clustering (SHCL) using SAM analysis revealed 587 differentially expressed genes in LMS compared to UPS, being 580 over-expressed and 7 down-expressed, but failed to discriminate between the neoplasms. However, over again the retroperitoneal LMS cases demonstrated similar expression profiles (Figure 2B). Only two LMS of the extremities (LMS14 and LMS15) clustered with the LMS-R samples. The IPA network analysis generated three main interactions networks (Table S2). The first network was associated with skeletal and muscular system development and function, tissue morphology, and cellular assembly and organization, including *ACTA2*, *MYLK*, *MYOCD* and *SRF* (Figure S1A). The second was related to cellular movement, cell morphology and cellular assembly and organization, and encompassed *ILK*, *CXCL1*, *LAMA5* and *SRC* (Figure S1B). The third network harbored genes associated with cell death, DNA replication, recombination, repair and gene expression, including *FLNA*, *MEF2C*, *CHEK1* and *BAD* (Figure S1C).

### Meta-analysis and protein-protein interaction network analysis

**Figure 3:**
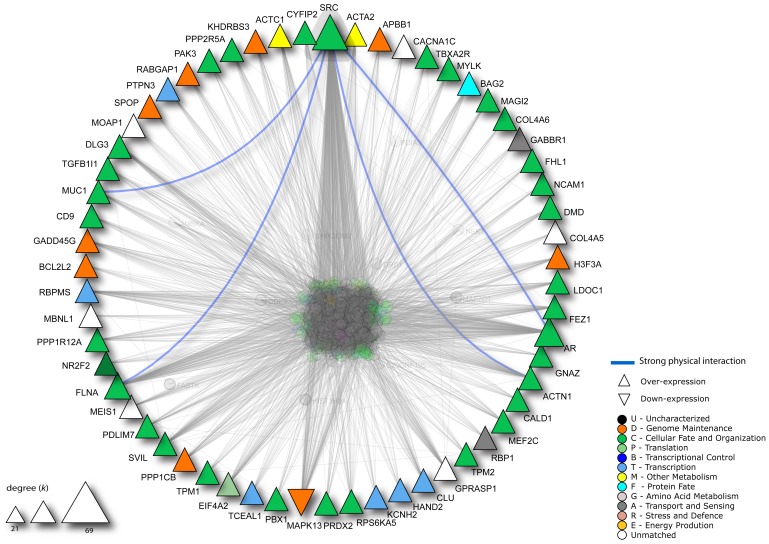
Protein-protein interaction network of 59 genes derived from the meta-analysis. Only proteins with more than 10 interactions partners were considered. The triangle size is proportional to the number of interactions for each protein. SRC presented the largest number of interactions, including four strong physical interactions found in the network. The interaction partners for each protein were obtained from the Interologous Interaction Database (I2D) 2.0 and the network was visualized and analyzed with the NAViGaTOR 2.3 software.

Aiming to validate the signature obtained in the comparison between LMS and UPS from the SAM analysis, a meta-analysis including five studies was conducted (Table 2) and revealed 316 of 587 differentially expressed genes obtained by comparison between LMS and UPS. A total of 193 differentially expressed genes (191 over-expressed and 2 down-expressed) in LMS compared with UPS exhibited the same expression pattern in all studies, including several genes related to muscular function (Table S3). Protein-protein interaction analysis involving the 193 validated meta-analysis genes revealed a network of 59 proteins with more than 10 interaction partners (Figure 3). SRC protein presented the highest number of interactions, in particular with ACTN1, AR, FLNA and MUC1.

**Table 2:** Soft-tissue sarcoma profiling studies included in the meta-analysis.

GEO accession	Study	Platform	Samples	Tumors included in the current study
GSE3443	Nielsen et al. [12]	Custom cDNA array	46	11 LMS vs. 6 UPS (MFH)
GSE2719	Detwiller et al. [25]	Affymetrix HG-U133A	54	6 LMS vs. 9 UPS (MFH)
GSE6481	Nakayama et al. [13]	Affymetrix HG-U133A	105	6 LMS vs. 21 UPS (MFH)
GSE21122	Barretina et al. [26]	Affymetrix HG-U133A	158	26 LMS vs. 3 UPS (pleomorphic MFH)
GSE21050	Chibon et al. [17]	Affymetrix HG-U133A Plus 2	310	85 LMS vs.136 UPS

GEO: Gene Expression Omnibus; LMS: Leiomyosarcoma; MFH: malignant fibrous histiocytoma; UPS: undifferentiated pleomorphic sarcoma.

### Real-time quantitative RT-PCR of *BAD, SRC, SRF*, and *MYOCD*

Based on the gene signature, gene network analysis, gene function and meta-analysis, *BAD*, *SRC*, *SRF* and *MYOCD* were selected to be further investigated by RT-qPCR. *SRF*, *SRC* and *BAD* were detected as central node genes in the main networks generated by IPA software (Figure S1), supporting their potential involvement in LMS development.

**Figure 4:**
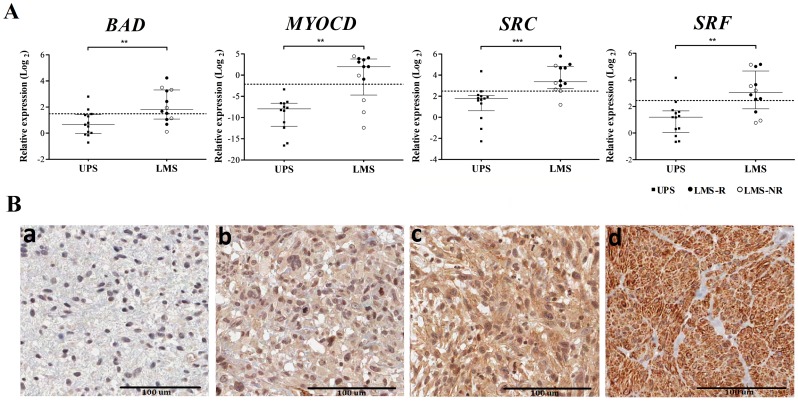
RT-qPCR of four genes and SRC protein expression by immunohistochemistry. (A) Dotplot showing the relative expression of the genes evaluated by RT-qPCR in 13 UPS and 12 LMS cases. For MYOCD gene 12 UPS and 12 LMS were evaluated. The dashed line represents an optimized threshold from ROC curves to discriminate LMS from UPS (**P<0.01; *** P<0.001) (Mann-Whitney U test). Error bars represent the median and interquartile range. (B) Examples of SRC immunostaining: negative (a); weak (b); moderate (c) and intense (d). Scores 0 and 1 were defined as SRC negative expression and scores 2 and 3 were considered SRC positive expression. Scale bars at 100 µm. Images captured with Scanscope XT Scanner System (Aperio Technologies, Inc., Vista, CA, EUA).

Similarly to the gene expression microarray, all genes showed significant over-expression (*P*<0.01) in LMS when compared to UPS (Figure 4A). Although not significant, *MYOCD* presented over-expression in LMS-R compared to non-retroperitoneal LMS (LMS-NR) (*P* = 0.123). Of all genes tested, *SRC* was revealed to be the most valuable discriminator for distinguishing between LMS and UPS (area under the ROC curve, AUC = 0.897 and accuracy = 92.0%) (Table S4). Two samples were classified incorrectly (LMS24 and UPS8) when using SRC as the discriminator.

### SRC protein expression by immunohistochemistry

SRC protein revealed negative/weak immunostaining (score 0–1) in 30 LMS and 36 UPS while positivity (scores 2–3) was detected in 22 LMS and two UPS (*P*<0.001) (Figure 4B). Although most LMS-R presented positivity for SRC (5/7), no significant difference was observed between LMS-R and LMS-NR (Table 3). SRC positivity exhibited high specificity (94.7%) and a positive predictive value of 91.7% in discriminating LMS from UPS.

**Table 3:** SRC protein expression levels evaluated by immunohistochemistry in 52 LMS and 38 UPS.

		Immunostaining					
Cases	Total	Negative Expression		Positive Expression		*P*	*P*
		Score 0	Score 1	Score 2	Score 3	(UPS vs. LMS)*	(LMS-R vs. LMS-NR)#
**UPS-NR**	32 (100%)	25 (78.1%)	5 (15.7%)	1 (3.1%)	1 (3.1%)		
**UPS-R**	6 (100%)	6 (100%)	0 (0%)	0 (0%)	0 (0%)	<0.001	0.216
**LMS-NR**	45 (100%)	22 (48.9%)	6 (13.3%)	3 (6.7%)	14 (31.1%)		
**LMS-R**	7 (100%)	2 (28.6%)	0 (0%)	0 (0%)	5 (71.4%)		

UPS: undifferentiated pleomorphic sarcoma; LMS: leiomyosarcoma; UPS-R: retroperitoneal UPS; UPS-NR: non-retroperitoneal UPS (including head and neck, trunk and extremity); LMS-R: retroperitoneal LMS; LMS-NR: non-retroperitoneal LMS (including head and neck, trunk and extremity);

*Chi-square test;

# Fisher exact test.

## Discussion

Large-scale gene expression studies in STS have many limitations, including evaluation of only a small number of fresh-frozen samples, a large number of histological subtypes included within the same report, a small number with the same histological subtype and the inclusion of cases treated with chemotherapy or radiotherapy prior to sample collection [3], [31]. Overall, these limitations reduce the probability of identifying recurrent genetic alterations with clinical significance. In this study, 22 UPS and 22 LMS samples were collected prior to treatment with chemotherapy or radiotherapy, with their diagnosis confirmed using the markers recommended in the literature.

Although the UHCL analysis was not able to accurately discriminate LMS from UPS, a distinct molecular profile for LMS-R was established, independent of histological grade (cluster I). Cluster II was divided in two subgroups and included the high-grade LMS-NR and UPS from different anatomical sites. It is important to highlight that one patient developed two UPS (UPS8 and UPS18), which clustered together and demonstrated a similar gene expression profile, suggesting that these samples were not two primary tumors as previously diagnosed. Comparison of the clusters with the clinicopathological findings and overall survival revealed no significant difference, probably due to the fact that most of the tumors presented with a high histological grade and similar prognosis.

Several reports have shown a strong similarity between UPS and LMS, suggesting that they share common oncogenic pathways and may correspond to different stages of the same tumor entity [9], [14], [15]. However, this study has demonstrated that LMS-R clusters separately. Both UHCL and SHCL analysis suggested that high-grade LMS-NR demonstrates a gene expression profile with a closer proximity to UPS than to LMS-R. Almost all genes that distinguished LMS-R from other sarcomas were over-expressed (580 of 587 genes).

The function and interaction network analysis revealed a large number of genes associated with muscle structure and function, including *ACTG2*, *CALD1*, *DMD*, *MYOCD*, *MYLK*, *SRF* and *TAGLN*. A meta-analysis with datasets from five studies supported these findings. In all studies, 193 of the 316 genes evaluated were over-expressed in LMS compared to UPS. A considerable number of genes related to muscle function were found to be over-expressed in all studies, including *ACTC1*, *ACTA2*, *ACTN1*, *CALD1*, *CNN1*, *MYLK*, *MYL9*, *DMD*, *SGCA*, *TAGLN*, *TPM1*, *TPM2* and *SMTN*.

In LMS, over-expression of genes associated with muscle structure has been previously reported in the literature [12], [14], [16], [32]. Recently, Italiano et al. [18] used genomic and transcriptomic analysis for 73 LMS and described a distinct clinical and molecular profile for LMS-R compared to LMS of the extremities. This finding was based mainly on over-expression of genes associated to muscle differentiation. MYOCD, a transcriptional cofactor of SRF, was found to be associated with muscular differentiation in well-differentiated retroperitoneal LMS [14], [18], [32].

In this study, overexpression of *MYOCD, SRF, BAD* and *SRC* in LMS was found, predominantly in LMS-R. These genes were tested for their potential as diagnostic markers to distinguish LMS from UPS. Combined accuracy, sensitivity and specificity were calculated for these genes, and *SRC* was identified as the most valuable discriminator. In addition, a higher level of SRC protein expression was detected in both LMS-R and LMS-NR when compared with UPS. Despite the use of SRC as a unique marker not being highly sensitive (<50%), its high specificity and positive predictive value (>90%) supports its potential use when in combination with other conventional muscular markers. It is important to highlight that *in silico* analysis of protein-protein interaction using the genes obtained in the meta-analysis showed a large number of interactions involving SRC, therefore suggesting its important role in the pathogenesis of LMS.

## Conclusions

A distinct molecular profile was demonstrated for LMS-R, mediated by over-expression of genes involved in muscular development and function. Both well-differentiated LMS-R [14], [20] and high grade LMS-R presented over-expression of *MYOCD*. Meta-analysis confirmed the involvement of 193 differentially expressed genes in the comparison between LMS and UPS. Our findings suggested that LMS-R is a new molecular entity of sarcomas whose alterations could be useful to stratify patients on specific therapeutic protocols. In addition, *in-silico* analysis revealed *SRC* as a central gene associated with LMS. Furthermore, SRC protein expression could be useful as a diagnostic marker to differentiate between LMS and UPS, especially in cases where muscular markers are negative.

## Supporting Information

Figure S1 **Graphic representation of the three interaction networks with the genes over-expressed (red) and down-expressed (green) in LMS compared to UPS.** Genes were associated with skeletal and muscular system development and function, tissue morphology, cellular assembly and organization in first network (A); related to cellular movement, cell morphology and cellular assembly and organization in second network (B); and associated with cell death, DNA replication, recombination, repair and gene expression in third network (C). The red and green colors tones are proportional to intensity of expression for each gene. The genes selected for validation are indicated in blue circles. Image adapted from Ingenuity Pathway Analysis (IPA) software.
(TIF)

Table S1 Primer sequences and properties of the transcripts evaluated by RT-qPCR.
(XLSX)

Table S2 Description of differentially expressed genes in LMS and UPS categorized in three main interaction networks according to IPA software.
(XLSX)

Table S3 Meta-analysis of 316 genes from the supervised analysis (LMS vs. UPS) present in five array datasets.
(XLSX)

Table S4 Accuracy, specificity, sensitivity and confidence interval analysis used to evaluate the potential of the genes tested, individually or in association, as diagnostic markers in LMS and UPS.
(XLSX)

Text S1 **Diagnostic criteria and IHC antibody panel used to define LMS and UPS.**
(DOCX)
