# Supplementary figures and images for: Gene Expression Profiling in Leiomyosarcomas and Undifferentiated Pleomorphic Sarcomas: SRC as a New Diagnostic Marker
Source: PLoS One. 2014 Jul 16;9(7):e102281. doi: 10.1371/journal.pone.0102281 (PMC4100821; doi:10.1371/journal.pone.0102281)

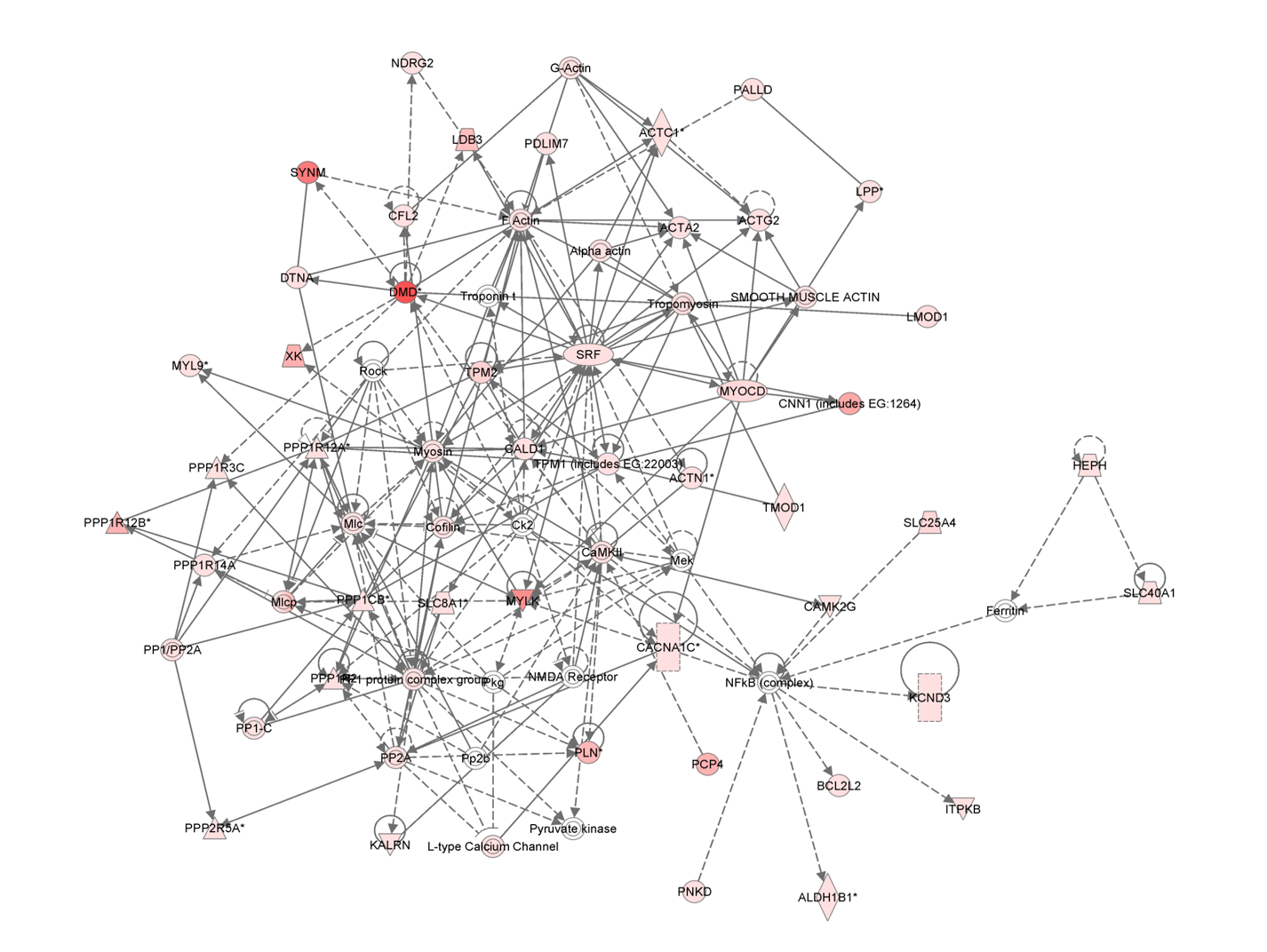

Supplement: Figure S1 — Graphic representation of the three interaction networks with the genes over-expressed (red) and down-expressed (green) in LMS compared to UPS. Genes were associated with skeletal and muscular system development and function, tissue morphology, cellular assembly and organization in first network (A); related to cellular movement, cell morphology and cellular assembly and organization in second network (B); and associated with cell death, DNA replication, recombination, repair and gene expression in third network (C). The red and green colors tones are proportional to intensity of expression for each gene. The genes selected for validation are indicated in blue circles. Image adapted from Ingenuity Pathway Analysis (IPA) software. (TIF) [file pone.0102281.s001.tif]
