# Supplementary material for: Gene Expression Profiling in Leiomyosarcomas and Undifferentiated Pleomorphic Sarcomas: SRC as a New Diagnostic Marker
Source: PLoS One. 2014 Jul 16;9(7):e102281. doi: 10.1371/journal.pone.0102281 (PMC4100821; doi:10.1371/journal.pone.0102281)
Supplement: Text S1 — Diagnostic criteria and IHC antibody panel used to define LMS and UPS. (DOCX) [file pone.0102281.s006.docx]

The IHC antibody panel used to confirm or exclude the diagnosis of LMS and UPS included SMA (Cell Marque, clone 1A4), Desmin (Ventana, clone DER11), HHF35 (Cell Marque, clone HHF 35), Caldesmon (Dako, clone h-CD), CD34 (Ventana, clone QBEnd 10), CD31 (Ventana, clone JC70), CD99 (Ventana, clone 0.13), S100 (Ventana, clone PAB), NSE (Ventana, clone E27), AE1/AE3 (Ventana, pool), EMA (Ventana, clone E29), HMB45 (Ventana, clone HMB45), MART-1/MelanA (Ventana, clone A-103), CD45 (LCA) (Ventana, clone RP2/18), CD63 (Cell Marque, clone NK1/C3), and MDM-2 (Neomarkers, polyclonal). Tumors showing pleomorphic morphology, negativity for all the markers tested or presenting focal expression of muscle markers (SMA, HHF35, desmin, and/or h-caldesmon) were considered as UPS. Tumors with spindle cell morphology and diffuse labelling of muscle markers were considered as LMS. Cases showing both spindle cell morphology and pleomorphic morphology, in addition to strong or diffuse labelling of muscle markers were also considered to be LMS. The diagnosis of undifferentiated retroperitoneal liposarcoma was excluded based on morphology (absence of specific lipomatous component), MDM2 immunostaining pattern, and evaluation of *MDM2* amplification by Fluorescence *in situ* Hybridization (FISH), as previously reported by Silveira et al. [13].
